# A quantitative fluorescence‐based approach to study mitochondrial protein import

**DOI:** 10.15252/embr.202255760

**Published:** 2023-03-20

**Authors:** Naintara Jain, Ridhima Gomkale, Olaf Bernhard, Peter Rehling, Luis Daniel Cruz‐Zaragoza

**Affiliations:** ^1^ Department of Cellular Biochemistry University Medical Center Göttingen Göttingen Germany; ^2^ Cluster of Excellence “Multiscale Bioimaging: from Molecular Machines to Networks of Excitable Cells” (MBExC) University of Göttingen Göttingen Germany; ^3^ Max Planck Institute for Multidisciplinary Science Göttingen Germany

**Keywords:** fluorescent precursor, *in vitro* import, mitochondria, presequence pathway, protein import, Methods & Resources, Membranes & Trafficking

## Abstract

Mitochondria play central roles in cellular energy production and metabolism. Most proteins required to carry out these functions are synthesized in the cytosol and imported into mitochondria. A growing number of metabolic disorders arising from mitochondrial dysfunction can be traced to errors in mitochondrial protein import. The mechanisms underlying the import of precursor proteins are commonly studied using radioactively labeled precursor proteins imported into purified mitochondria. Here, we establish a fluorescence‐based import assay to analyze protein import into mitochondria. We show that fluorescently labeled precursors enable import analysis with similar sensitivity to those using radioactive precursors, yet they provide the advantage of quantifying import with picomole resolution. We adapted the import assay to a 96‐well plate format allowing for fast analysis in a screening‐compatible format. Moreover, we show that fluorescently labeled precursors can be used to monitor the assembly of the F_1_F_0_ ATP synthase in purified mitochondria. Thus, we provide a sensitive fluorescence‐based import assay that enables quantitative and fast import analysis.

## Graphical abstract

This study presents a non‐radioactive mitochondrial import assay for matrix proteins using import substrates conjugated to a fluorophore.

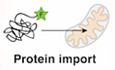

## Introduction

Mitochondria play central roles in cellular metabolism and signaling processes (Nunnari & Suomalainen, 2012). While mitochondria possess a small genome, most mitochondrial proteins are nuclear‐encoded and imported after their synthesis in the cytosol (Neupert & Herrmann, 2007; Richter‐Dennerlein *et al*, 2015; Pfanner *et al*, 2019; Araiso *et al*, 2022). The mitochondrial proteome comprises more than 1,000 different proteins in the yeast *Saccharomyces cerevisiae* (Sickmann *et al*, 2003; Morgenstern *et al*, 2017; Wiedemann & Pfanner, 2017; Di Bartolomeo *et al*, 2020). The translocation of the precursor proteins across the outer and inner mitochondrial membranes requires multi‐subunit protein translocation machineries in the membranes. The TOM (translocase of the outer membrane) complex facilitates translocation through the outer membrane, while TIM (translocase of the inner membrane) complexes mediate the translocation of precursors across the inner membrane (Berthold *et al*, 1995; Lill & Neupert, 1996; Wiedemann & Pfanner, 2017; Araiso *et al*, 2022). The majority of precursor proteins are directed across both membranes by N‐terminal presequences, consisting of amphipathic alpha helices, which are recognized by receptors in the TOM and TIM23 complex (Roise *et al*, 1986; Brix *et al*, 1997; Geissler *et al*, 2002; Neupert & Herrmann, 2007; Yamano *et al*, 2008; Chacinska *et al*, 2009; Vögtle *et al*, 2009; Yamamoto *et al*, 2009; Schulz *et al*, 2011; Araiso *et al*, 2022). The import by the TIM23 complex into the matrix requires membrane potential across the inner membrane (Δψ) and the activity of the presequence translocase‐associated motor (PAM) complex. Upon import into the matrix, the presequence is cleaved by the mitochondrial processing peptidase (MPP; Mossmann *et al*, 2012; Schulz *et al*, 2015; Wiedemann & Pfanner, 2017).

Import into mitochondria is commonly studied by utilizing an *in vitro* import assay in which [^35^S]‐labeled precursor proteins are imported post‐translationally into isolated mitochondria (Harmey *et al*, 1977; Maccecchini *et al*, 1979). For this, radiolabeled precursors are synthesized in reticulocyte lysates and incubated with purified mitochondria. Dissipation of the membrane potential by inhibitors of the OXPHOS system and uncouplers is used to block import. Protease treatment of the reaction following import removes non‐imported proteins from the system. Samples are analyzed by SDS– or BN‐PAGE, and proteins are visualized by autoradiography. This *in vitro* import assay has been instrumental in dissecting the mechanisms of protein translocation across the mitochondrial membrane as it provides high sensitivity and kinetic resolution. However, absolute quantitative information on the imported amounts of precursors is challenging to obtain in this setup, and the use of isotopes requires special safety precautions that are not readily available to all researchers. Moreover, the radioactive approach is difficult to combine with high‐throughput screening approaches.

Here we report on a fluorescence‐based method to monitor *in vitro* mitochondrial protein import using precursor‐fluorophore fusion protein as a substrate. The non‐radioactive method is sensitive and fast and allows working with chemical quantities of import‐competent protein. The fluorescent approach provides the advantage of a fully quantitative output with picomolar resolution and the potential to perform import in a plate format for rapid results. We show that, in addition to monitoring protein import, fluorescently labeled proteins can also be utilized to analyze the assembly of protein complexes in purified mitochondria.

## Results and Discussion

### Jac1_488_ fluorescent precursor enables quantitative import analysis

**Figure 1:**
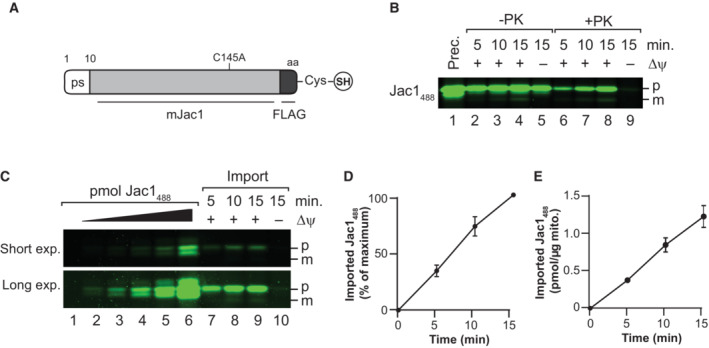
Import of fluorescent precursor into mitochondria Schematic presentation of the modified Jac1 protein with C‐terminal cysteine for fluorophore addition.Jac1_488_ was imported into purified mitochondria for indicated times and treated with Proteinase K (PK) or left untreated as indicated. For comparison, 47 pmol of precursor was loaded (lane 1). Prec., purified precursor protein; p, precursor; m, mature protein; Δψ, membrane potential.Jac1_488_ import into mitochondria as in (B), right side; Jac1_488_ protein dilution series, left side.Quantification of import of Jac1_488_ (maximal signal at 15 min import time, 100%); error bars indicate (SEM; *n* = 3).Quantification of absolute imported amounts in picomoles Jac1_488_ per μg of mitochondria; error bars indicate SEM (*n* = 3). Data information: The values represented in the graphs correspond to the arithmetic mean ± standard error of the mean (SEM). The number of biological independent replicates (*n*) for each experiment is indicated for each assay.

**Figure EV1:**
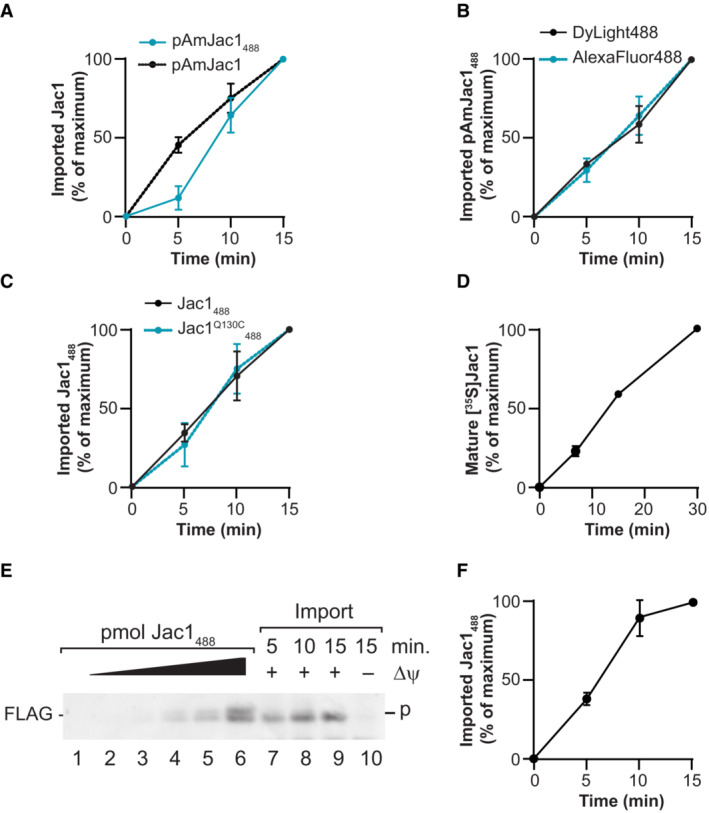
Effect of precursor modification with a fluorophore on protein import Quantification of import of pAmJac1 modified (pAmJac1_488_) and non‐modified (pAmJac1). The amount of imported protease‐protected samples at 15 min was set to 100% in each case; error bars indicate SEM (*n* = 4).Quantification of import of pAmJac1 conjugated to DyLight488 or Alexa Fluor 488. The amount of imported protease‐protected samples at 15 min was set to 100% in each case; error bars indicate SEM (*n* = 3).Quantification of import of Jac1 modified with DyLight488 at the C‐terminus (Jac1_488_) and internally (Jac1^Q130C^_488_). The amount of imported protease‐protected samples at 15 min was set to 100% in each case; error bars indicate SEM (*n* = 3).Quantification of import of radioactive [^35^S]Jac1. The amount of imported protease‐protected samples at 30 min was set to 100%; error bars indicate SEM (*n* = 3).Immunoblot‐based analysis of Jac1_488_ import into mitochondria from Fig 1C. The PDVF membranes were incubated with anti‐FLAG antibody for Jac1 detection.Quantification by immunoblot‐based detection of Jac1_488_ import shown in (E). The amount of imported protease‐protected samples at 15 min was set to 100%; error bars indicate SEM (*n* = 3). Data information: The values represented in the graphs correspond to the arithmetic mean ± standard error of the mean (SEM). The number of biological independent replicates (*n*) for each experiment is indicated for each assay.

Based on the previous observation that a fluorescently labeled precursor protein retains the ability to be imported into mitochondria (Cruz‐Zaragoza *et al*, 2021), we set out to establish a non‐radioactive standard import assay. For the initial set of experiments, a construct consisting of the *S. cerevisiae* Jac1 protein with its authentic N‐terminal presequence fused to a C‐terminal FLAG tag was used (Fig 1A). The Jac1 used here carried a C145A exchange and an additional cysteine residue at the C‐terminal, allowing for adding a fluorophore. For purification of the construct from *E. coli*, the protein carried a His‐tag, which was cleaved off post‐purification through a flanking SUMO protease site, preserving the N‐terminus of the protein. After purification, the Jac1 fusion protein was modified by the maleimide‐mediated addition of a DyLight fluorophore to the terminal cysteine residue (Jac1_488_). Next, we imported the precursor into isolated mitochondria. Samples were split after the import reaction and treated with Proteinase K (PK) to remove non‐imported precursor. As a negative control, the membrane potential was dissipated prior to import. Samples were subjected to SDS–PAGE, and gels were scanned at the DyLight fluorophore emission range using a fluorescence scanner (Fig 1B). The Jac1_488_ precursor was imported into mitochondria in a time and membrane potential‐dependent manner, as apparent in the protease‐treated samples (Fig 1B). The presence of the fluorophore did not impact the protein import capacity, as shown by importing the unmodified protein followed by immunodetection of the FLAG‐tag (Fig EV1A). Import kinetics were independent of the fluorophore's hydrophobicity as determined by comparing the import of a protein modified with DyLight488 (a fluorophore hydrophobic) to that modified with Alexa Fluor 488 (a more hydrophilic fluorophore; Fig EV1B; Hughes *et al*, 2014). In addition, a protein with an internal cysteine modified conjugated to a fluorophore (Jac1^Q130C^_488_) showed similar kinetics upon import compared to the C‐terminally modified protein (Fig EV1C). Interestingly, Jac1_488_ did not display efficient processing upon import. Based on results from import assays of other protein constructs tested during this study, this is not a general phenomenon observed for fluorescent precursors but specific to this protein and its presequence.

After confirming that the fluorescent substrate was imported efficiently into mitochondria, we aimed to obtain quantitative data on the imported protein amounts. To this end, dilutions of the purified precursor protein were used as a standard and loaded together with the import samples on the gel (Fig 1C). A titration curve of the precursor standard was plotted to determine the absolute amount of imported protein per μg of mitochondria. We calculated that about 72 fmol protein was imported per minute per μg mitochondria (Fig 1E). In this timeframe, the import reaction was still in the linear range (Fig 1D).

The use of radioactive precursors is considered as the “gold standard” in the field of mitochondrial protein import. To compare both methods, radioactive [^35^S]Jac1 was imported under the same conditions as Jac1_488_. The import kinetics of [^35^S]Jac1 was similar to that of the fluorescent Jac1_488_, where no saturation was reached after 30 min (Fig EV1D). In addition, addressing if the use of fluorescence detection represented an advantage over detection by an immunoblot‐based approach, we immunodetected the imported Jac1_488_ (corresponding to Fig 1C) with anti‐FLAG antibody (Fig EV1E and F). In these experiments, the fluorescence detection was linear over a longer range than in the Western blot approach that is likely to be limited by antibody binding to the cognate epitope.

We concluded that fluorescently labeled precursors can be used for *in vitro* import and that the assay allowed us to obtain quantitative data on mitochondrial import. Accordingly, an absolute comparison between different substrates and import conditions can be obtained.

### Jac1_488_ enables functional analysis of the import machinery

**Figure 2:**
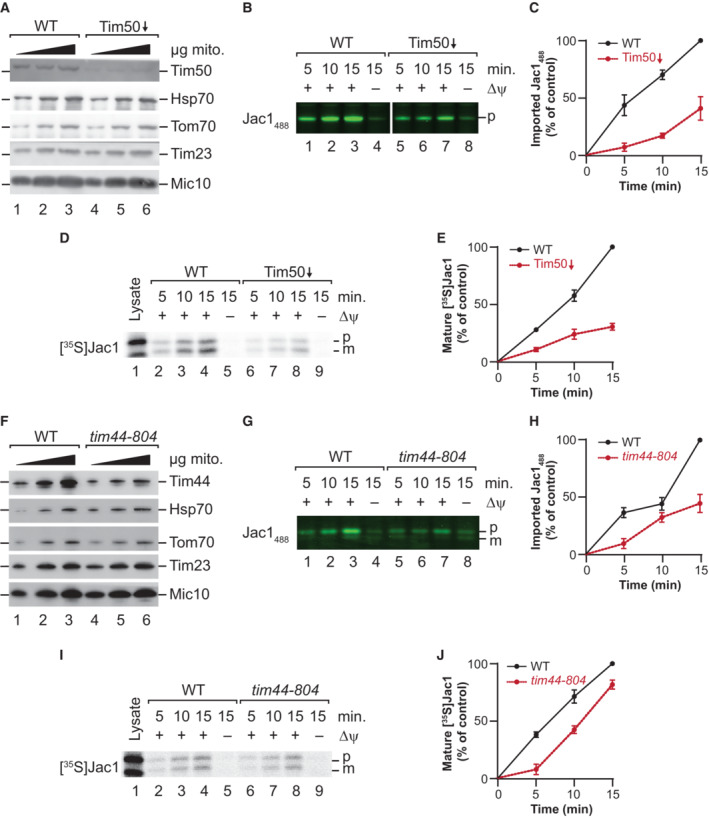
Import into mutant mitochondria Steady‐state protein levels in wild‐type (WT) and Tim50‐depleted mitochondria visualized by Western blotting and immunodetection with indicated antisera.Jac1_488_ was imported into wild‐type (WT) and Tim50‐depleted mitochondria for indicated times, and samples were treated with Proteinase K. p, precursor.Quantification of Jac1_488_ import into wild‐type (WT) and Tim50‐depleted mitochondria. The amount of imported protease‐protected protein in WT mitochondria at 15 min was set to 100%; error bars indicate SEM (*n* = 3).[^35^S]Jac1 was imported into wild‐type (WT) and Tim50‐depleted mitochondria for indicated times, and samples were treated with Proteinase K. p, precursor; m, mature.Quantification of [^35^S]Jac1 import into wild‐type (WT) and Tim50‐depleted mitochondria. The amount of imported protease‐protected protein in WT mitochondria at 15 min was set to 100%; error bars indicate SEM (*n* = 3).Steady‐state protein levels in wild‐type (WT) and *tim44‐804* mitochondria visualized by Western blotting and immunodetection with indicated antisera.Jac1_488_ was imported into wild‐type (WT) and *tim44‐804* mitochondria for indicated times, and samples were treated with Proteinase K. p, precursor; m, mature.Quantification of Jac1_488_ import into wild‐type (WT) and *tim44‐804* mitochondria. The amount of imported protease‐protected protein in WT mitochondria at 15 min was set to 100%; error bars indicate SEM (*n* = 3).[^35^S]Jac1 was imported into wild‐type (WT) and *tim44‐804* mitochondria for indicated times, and samples were treated with Proteinase K. p, precursor; m, mature.Quantification of [^35^S]Jac1 import into wild‐type (WT) and *tim44‐804* mitochondria. The amount of imported protease‐protected protein in WT mitochondria at 15 min was set to 100%; error bars indicate SEM (*n* = 3). Data information: The values represented in the graphs correspond to the arithmetic mean ± standard error of the mean (SEM). The number of biological independent replicates (*n*) for each experiment is indicated for each assay.

*In vitro* import into mitochondria is the key technology to dissect the mechanisms and components of protein translocation. To assess if the fluorescently‐labeled precursor could be used to analyze defects in protein transport, we imported Jac1_488_ into purified yeast mitochondria with defects in the import machineries. Therefore, a temperature‐sensitive mutant of *Tim44* (*tim44‐804*) and a yeast strain in which the *TIM50* gene was controlled by a GAL‐promotor, allowing to decrease steady‐state levels of Tim50, were selected (Geissler *et al*, 2002; Hutu *et al*, 2008). Tim50 is the essential, central presequence receptor of the TIM23 complex and is required for precursor import (Geissler *et al*, 2002; Yamamoto *et al*, 2002; Mokranjac *et al*, 2003; Qian *et al*, 2011; Schulz *et al*, 2011). Mitochondria depleted for Tim50 were isolated from *S. cerevisiae* (Schulz *et al*, 2011), and steady‐state protein levels were analyzed to confirm efficient knockdown. While the Tim50 levels were reduced in the mutant strain, Tom70, Tim23, and Hsp70 levels remained similar to the wild‐type (WT), indicating that the remaining import machinery constituents were not affected by the knockdown (Fig 2A). Jac1_488_ was imported into WT and Tim50‐depleted mitochondria (Fig 2B). As expected, Jac1_488_ import was severely affected in the mutant mitochondria, which amounted to about 40% of the WT import after 15 min (Fig 2C). The observed import defect matched the decrease in import observed with radioactively labeled Jac1 (Fig 2D and E).

Tim44 is a constituent of the mitochondrial import motor and is required for matrix protein transport (Blom *et al*, 1993; Schneider *et al*, 1994). We used a Tim44 temperature‐conditional yeast mutant strain (*tim44‐804*), which displays an import defect upon the shift of mitochondria to 37°C (Hutu *et al*, 2008). Western blot analysis of the protein steady‐state levels in *tim44‐804* displayed slightly reduced amounts of Tim44 in mitochondria, while other analyzed translocase constituents were not decreased in the mutant (Fig 2F). Import of Jac1_488_ into WT and *tim44‐804* mutant mitochondria showed a mutant‐specific decrease in import (Fig 2G and H). Interestingly, the reduction in import was less pronounced when assayed using the radiolabeled Jac1 (Fig 2I and J). The increased import defect observed for the fluorescently labeled precursor is possibly due to the larger quantities of precursor applied to mitochondria compared to the radiolabeled counterpart, which challenges the import machinery for translocation. It is also conceivable that chaperones associated with the radiolabeled precursor after synthesis in the reticulocyte lysate may stimulate the import by unfolding the precursor for import. In summary, these results confirm that import defects can be efficiently assayed using the fluorescently‐labeled precursor.

### Effect of presequences swapping on import efficiency

**Figure 3:**
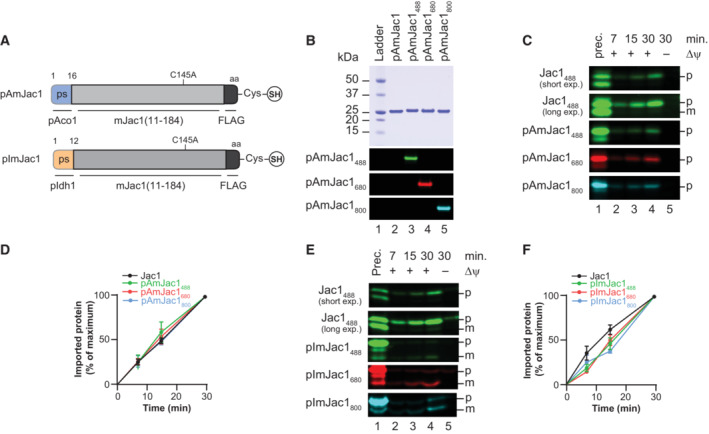
Import of precursor variants into mitochondria Schematic presentation of pAmJac1 and pImJac1, with presequences derived from Aco1 and Idh1, respectively, fused to the N‐terminus of the mature Jac1 portion.Purified pAmJac1 was conjugated with different fluorophores as indicated. Detected without bleed through under fluorescence imaging conditions.Jac1_488_ and pAmJac1 conjugated to DyLight 488, 680, and 800 were imported into wild‐type mitochondria for indicated times, and samples were treated with Proteinase K. Prec., purified precursor protein; p, precursor; m, mature.Quantification of Jac1_488_ and pAmJac1 conjugated to DyLight 488, 680, and 800 import into wild‐type (WT). The amount of imported protease‐protected samples at 30 min was set to 100%; error bars indicate SEM (*n* = 3).Jac1_488_ and pImJac1 conjugated to DyLight 488, 680, and 800 were imported into wild‐type mitochondria for indicated times and samples treated with Proteinase K. Prec., purified precursor protein; p, precursor; m, mature.Quantification of Jac1_488_ and pImJac1 conjugated to DyLight 488, 680, and 800 import into wild‐type (WT). The amount of imported protease‐protected samples at 30 min was set to 100%; error bars indicate SEM (*n* = 3). Data information: The values represented in the graphs correspond to the arithmetic mean ± standard error of the mean (SEM). The number of biological independent replicates (*n*) for each experiment is indicated for each assay.

**Figure EV2:**
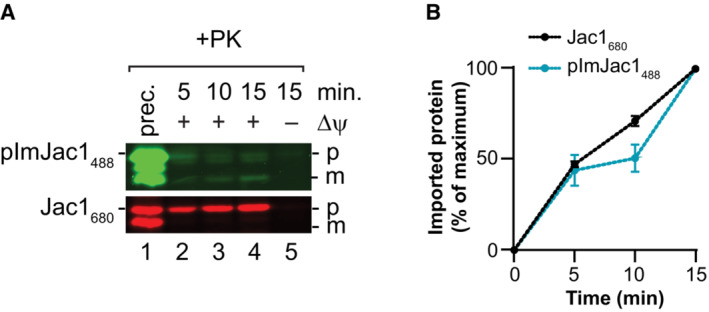
Parallelization of import Co‐import of Jac1_680_ and pImJac1_488_ into wild‐type mitochondria for indicated times. Samples were treated with Proteinase K. Prec., purified precursor protein; p, precursor; m, mature.Quantification of co‐import shown in (A). The amount of imported protease‐protected samples at 15 min was set to 100% in each case; error bars indicate SEM (*n* = 3). Data information: The values represented in the graphs correspond to the arithmetic mean ± standard error of the mean (SEM). The number of biological independent replicates (*n*) for each experiment is indicated for each assay.

To utilize this method to study different precursor characteristics, we synthesized Jac1 constructs with different targeting signals. For this, the authentic presequence of Jac1 was replaced with presequences of Idh1 (Isocitrate dehydrogenase 1) or Aco1 (Aconitase 1). These presequences were selected due to their similarity to the Jac1 presequence regarding length and charge (^1–10^Jac1^ps^, +2.26 net charge; ^1–12^Idh1^ps^, +2.27 net charge; ^1–16^Aco1^ps^, +3.27 net charge; Fig 3A). The same procedure was followed for the purification and modification of these constructs as described above for the authentic Jac1. All constructs were modified with three different DyLight fluorophores (with excitation wavelengths 488, 680, and 800 nm), assuming that the modification should not affect import but allow multiplex import using precursors with different fluorophores in the same sample (Fig 3B). Subsequently, all precursor constructs were imported into purified mitochondria using the Jac1_488_ precursor as a control. After import, samples were analyzed by SDS–PAGE, and the fluorescence signal of the imported proteins was quantified. All fusion proteins displayed membrane potential‐dependent import that increased with time (Fig 3C and E). Despite a slightly more positively charged and longer presequence in the case of the Aco1, the import of the pAmJac1 (mature Jac1 protein with Aco1 presequence) showed no significant difference in import compared to the Jac1 control (Fig 3D). Similar to Jac1, the pAmJac1 variant did not display efficient processing after import (Fig 3C). In the import experiments, pImJac1 (mature Jac1 protein with Idh1 presequence) precursor variants differed slightly compared to the control, despite the almost identical size and charge of the presequence (Fig 3F). However, upon import of pImJac1, we observed efficient processing of the Idh1 presequence (Fig 3E). These two examples showed that the import of fluorescently labeled precursors represents a means to analyze precursor properties for mitochondrial protein import. In both cases, the import of the constructs was not affected by choice of fluorophore (Fig 3D and F). Accordingly, different combinations of fluorophores can be used to monitor import. Since there was no fluorescence bleed‐through between different scanning channels, we asked whether using differently labeled precursors would enable multiplexing of import assays using different precursors tagged by different fluorophores. For this, we mixed pImJac1_488_ and Jac1_680_ and co‐imported them into mitochondria. By direct comparison to the precursor input (prec.), we could observe that the construct containing the original Jac1 presequence was imported more efficiently than the construct containing the Idh1 presequence (Fig EV2A). In contrast, the kinetic of the import reaction was similar in both cases (Fig EV2B).

### Assessing import in 96‐well plate format

**Figure 4:**
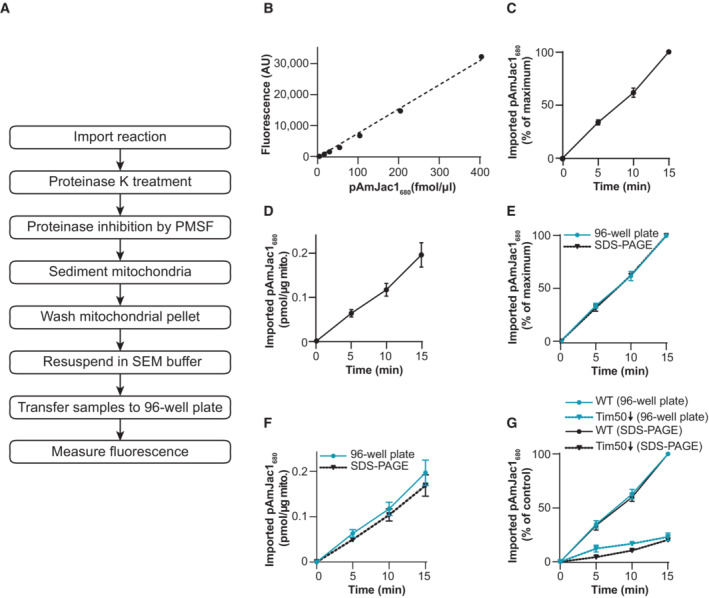
Analyzing import in a multi‐well plate format Workflow for transfer of imported samples into 96‐well plate for readout.Standard curve depicting fluorescence signal plotted against concentration of pAmJac1_680_ dilutions.Import of pAmJac1_680_ into wild‐type mitochondria. After import and proteinase K treatment, fluorescence was measured in 96‐well format. The amount of imported protease‐protected samples at 15 min was set to 100%; error bars indicate SEM (*n* = 8).Quantification of picomoles of pAmJac1_680_ imported per μg mitochondria as assessed in 96‐well format; error bars indicate SEM (*n* = 8).pAmJac1_680_ was imported into purified mitochondria, and samples were analyzed by SDS–PAGE or in 96‐well format. The amount of imported, protease‐protected samples at 15 min was set to 100% in each case; error bars indicate SEM (*n* = 8 for 96‐well plate analysis, *n* = 3 for SDS–PAGE).Comparison of absolute import of pAmJac1_680_ into wild‐type mitochondria quantified from 96‐well format and SDS–PAGE. Error bars indicate SEM (*n* = 8 for 96‐well plate analysis, *n* = 3 for SDS–PAGE).pAmJac1_680_ was imported into wild‐type (WT) and Tim50‐depleted mitochondria for indicated times and samples treated with proteinase K. Samples were analyzed in 96‐well format and by SDS–PAGE. The amount of imported protease‐protected protein in WT mitochondria at 15 min was set to 100%; error bars indicate SEM (*n* = 5 for 96‐well plate analysis, *n* = 4 for SDS–PAGE). Data information: The values represented in the graphs correspond to the arithmetic mean ± standard error of the mean (SEM). The number of biological independent replicates (*n*) for each experiment is indicated for each assay.

While the current standard import assay requires the separation of proteins by PAGE analysis to observe the labeled protein, we were curious if we could adapt the process to a plate format in which samples could be analyzed rapidly with a fluorescence plate reader. Therefore, we performed the import assay using the pAmJac1_680_ precursor as described above and subsequently treated the mitochondria with proteinase K. Following import, mitochondria were re‐isolated, resuspended, and transferred to 96‐well plates (Fig 4A). For comparison, the import reactions were split and analyzed in a plate assay and by SDS–PAGE. To enable quantification, a dilution series of the precursor was measured as a standard (Fig 4B). Fluorescence measurements of pAmJac1_680_ import reactions revealed a time‐dependent localization of the construct to mitochondria (Fig 4C). The fluorescence measured in the membrane potential depleted sample was subtracted from the individual measurements to correct for background binding. Quantification of the plate assay showed that 14 fmol of pAmJac1_680_ protein were imported per minute per μg mitochondria (Fig 4D). Accordingly, a plate format is suitable for assessing the import of labeled precursors, which can be quantified much faster than with a gel‐based system. A comparison between the results of the plate format and the standard PAGE analysis showed that both types of analysis provided similar data on the kinetics of the import reaction (Fig 4E). A quantitative comparison showed only a slight divergence between the two approaches that can possibly be attributed to precursor fragments detected by total fluorescence but not resolved on the gel (Fig 4F).

Next, we addressed if the plate format could be applied to analyze defects in mitochondrial import. For this, we performed import into wild‐type and Tim50‐depleted mitochondria (Fig 4G). Similar import kinetics were apparent with both approaches, and the magnitude of the mutant import defect was comparable. Accordingly, the analysis of import reactions by plate assay provided an efficient means for import studies. Yet, while the plate format saves time and provides accurate readouts on import kinetics and efficiency, it cannot provide information on protein processing after import and can only give a value for the total imported protease‐protected protein amounts.

### Fluorescent Atp5 enables analysis of complex assembly

**Figure 5:**
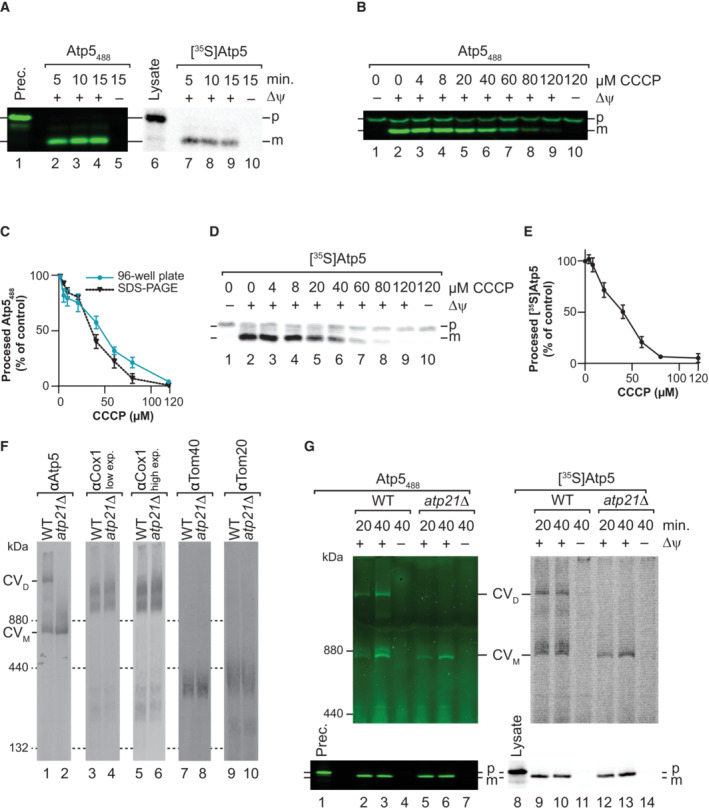
Fluorescence‐based protein assembly Atp5_488_ (left panel) or [^35^S]Atp5 (right panel) was imported into mitochondria for indicated times and in the presence or absence of a membrane potential (Δψ). After proteinase K treatment, samples were separated by SDS–PAGE and analyzed by fluorescence scanning or digital autoradiography. Prec., purified precursor protein; p, precursor; m, mature protein.Import of Atp5_488_ with increasing concentrations of CCCP to study dependency of import on membrane potential (Δψ). P, precursor; m, mature protein.Comparison of import of Atp5_488_ with increasing CCCP concentrations quantified from 96‐well format and SDS–PAGE. The amount of imported protease‐protected samples in the absence of CCCP was set to 100%; error bars indicate SEM (*n* = 4 for 96‐well plate analysis, *n* = 3 for SDS–PAGE).[^35^S]Atp5 was imported with increasing CCCP concentrations as described in (B). Samples were separated by SDS–PAGE and analyzed by digital autoradiography.Quantification of import of [^35^S]Atp5 with increasing CCCP concentrations. The amount of imported protease‐protected protein in the absence of CCCP was set to 100%; error bars indicate SEM (*n* = 3).BN‐PAGE steady‐state analysis of complex V, complex IV, and TOM in mitochondria isolated from WT and *atp21*Δ yeast strains.Atp5_488_ (left panel) or [^35^S]Atp5 (right panel) was imported into wild‐type and *atp21*Δ mitochondria for indicated times and in the presence or absence of a membrane potential (Δψ). After proteinase K treatment, samples were solubilized in digitonin buffer and separated by BN‐PAGE. Aliquots of the samples were analyzed by SDS–PAGE (lower panels). Subsequently, proteins were visualized by fluorescence scanning or digital autoradiography. Complex V dimer (CV_D_) and monomer (CV_M_). Prec., purified precursor; p, precursor; m, mature protein. Data information: The values represented in the graphs correspond to the arithmetic mean ± standard error of the mean (SEM). The number of biological independent replicates (*n*) for each experiment is indicated for each assay.

**Figure EV3:**
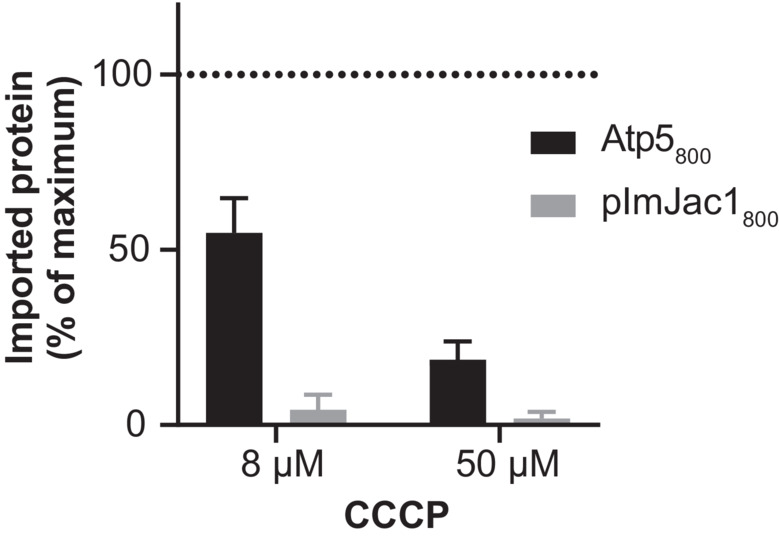
Membrane potential influence over the import of different precursors Quantification of import of Atp5_800_ and pImJac1_800_ over 15 min into mitochondria treated with 8 and 50 μM CCCP. The amount of imported protease‐protected protein in the absence of CCCP was set to 100%; error bars indicate SEM (*n* = 3). Data information: The values represented in the graphs correspond to the arithmetic mean ± standard error of the mean (SEM). The number of biological independent replicates (*n*) for each experiment is indicated for each assay.

During the study, we tested various protein‐fluorophore fusion constructs. Among these was Atp5_488_, a subunit of the F_1_F_0_ ATP synthase, which displayed efficient processing comparable to that seen in the autoradiograph of import reactions using a radioactive Atp5 version (Fig 5A). The low background and strong signal suggested that it could be a good candidate for the 96‐well plate import readout. Hence, we used this precursor to perform further import experiments and to corroborate the results with the import of radioactive Atp5. We tested the dependence of Atp5 on the mitochondrial membrane potential for import using the uncoupler CCCP (carbonyl cyanide‐m‐chlorophenylhydrazone) that allows the dissipation of the Δψ in a concentration‐dependent manner (Martin *et al*, 1991). To this end, we titrated increasing amounts of CCCP and analyzed protein import into mitochondria. We observed that the amount of mature Atp5_488_ decreased with increasing CCCP concentration (Fig 5B). We compared the decrease of import with increasing CCCP concentrations between the fluorescent Atp5_488_ protein quantified using a gel system and a 96‐well plate format (Fig 5B and C) and [^35^S]Atp5 analyzed by autoradiography (Fig 5D and E). In all three cases, a similar Δψ‐dependence of the import was observed, supporting the usefulness of the fluorescence approach for analyzing the bioenergetics of protein import. Notably, we could detect different sensitivity towards changes in membrane potential of protein import for precursors with different presequences such as Atp5_800_ and pImJac1_800_ (Fig EV3).

The availability of a subunit of an OXPHOS complex suitable for protein import led us to ask if a fluorescence import approach was appropriate for the analysis of OXPHOS complex assembly. To address this, we purified mitochondria from an *atp21*Δ strain that lacks the ability to form complex V dimers, as can be analyzed by Blue Native PAGE (Fig 5F, lanes 1–2). Radiolabeled Atp5 and Atp5_488_ efficiently assembled into monomeric and dimeric ATP synthase in wild‐type mitochondria in a membrane potential‐dependent manner. In *atp21*Δ mutant mitochondria, both proteins are only assembled into the monomeric ATP synthase as these mitochondria lack the dimeric form (Fig 5G). Accordingly, the OXPHOS protein complex assembly can be studied with a fluorescent fusion protein. The non‐radioactive approach provides similar information and sensitivity as the radioactive assay.

### Advantages and limitations of fluorescence‐based *in vitro* import

Here, we present a new approach to studying protein import into mitochondria. The commonly used method in the field is the synthesis of precursor proteins by *in vitro* translation using radioactive labeling for detection after protein or protein complex separation by PAGE analysis. Yet, using radioactive substances is problematic for many labs due to safety considerations related to the technique. Different approaches have been previously attempted to address protein import with non‐radioactive strategies. In principle, the use of recombinant proteins is established and has the advantage of providing import substrates in chemical and translocase saturating quantities with Western blot‐based detection (Voos *et al*, 1993; Mokranjac *et al*, 2005; Schulz & Rehling, 2014). In a recent study, Pereira *et al* (2019) established an approach utilizing a bipartite luminescence system that employs NanoBiT—a split luciferase—to monitor protein import. Yet, this approach requires introducing one of the components in advance of the import assay into mitochondria making it less flexible to use in a mutant context.

Here, we provide a non‐radioactive alternative based on the use of fluorescently labeled proteins and complementary to the existing strategies, filling the analytic gaps of the current techniques. Chemical modification with fluorescent dyes allows the use of gel‐based and multi‐well plate‐based approaches. Moreover, as the absolute amounts of precursors can be determined, it is possible to assess the protein import quantitatively. Finally, the in‐gel and 96‐well formats offer a faster detection mode than radioactive assay. Beyond these applications, we show that after the fluorescently labeled protein import, it can assemble into its target complex. Hence, the *in vitro* import system allows analysis of assembly processes similar to radiolabeled proteins and broadens the scope of non‐radioactive approaches. Yet, in its current form, the approach comes with a limitation. At this stage, the fluorescence‐based import assay has been established for the import of soluble proteins entering the matrix via the TIM23 presequence pathway. The study of other import pathways requires the purification and modification of different classes of precursor proteins. For example, the TIM22 pathway substrates are extremely hydrophobic proteins, the purification and modification of which are challenging. Yet, modification of such precursors in urea buffer and subsequent import appears to be a feasible option. Further, substrates imported to the intermembrane space (IMS) through the MIA pathway require free cysteine residues to form transient disulfide bonds for receptor recognition. Although this imposes restrictions on the use of maleimide‐based chemical modification, other labeling methods could be used instead (e.g., NHS‐based). Moreover, as seen for Jac1, in special cases, recombinant proteins might not provide information regarding protein processing. However, a fluorescence‐based import system remains advantageous due to the absence of radioactivity, faster processing time, multiplexed precursor import, fully quantitative readout, and it can be used for 96‐well plate‐based fast measurement.

## Materials and Methods

### Plasmid generation for protein expression in bacteria

For the production of recombinant Jac1‐FLAG‐Cys, a plasmid reported previously was used (Cruz‐Zaragoza *et al*, 2021) where Jac1 is tagged at the N‐terminus with a His_14_‐SUMO‐tag. To swap the Jac1 presequence, the sequence encoding presequences of *S. cerevisiae* Aco1 and Idh1 were added by PCR at the 5′ of Jac1 ORF, removing the Jac1 presequence, followed by Gibson assembly. *ATP5* gene was amplified from *S. cerevisiae* genomic DNA and ligated into pJET vector using CloneJET PCR Cloning kit (Thermo Scientific). Site‐directed mutagenesis was used to recode the cysteine at position 117 to alanine. The expression vector was amplified by PCR while removing Jac1 ORF but preserving FLAG‐tag and C‐terminal cysteine coding sequence. Finally, the mutant ORF was amplified by PCR and inserted in frame after the His_14_‐SUMO‐tag sequence from the amplified vector to generate a plasmid for the expression of His_14_‐SUMO‐Atp5^C117A^‐FLAG‐Cys.

### Recombinant protein expression and purification

Recombinant Jac1 its variants with different presequences (pAmJac1 and pImJac1) and Atp5^C117A^ were purified as follows. Plasmids were transformed into *Escherichia coli* BL21 Tuner (DE3) strain (Sigma‐Aldrich) or Rosetta DE3 (Novagen) in the case of Atp5^C117A^. One colony was inoculated into LB medium supplemented with 2% glucose and 50 μg/ml kanamycin. The preculture was incubated for 8 h at 30°C. The OD_600nm_ was determined, and fresh medium was inoculated at initial OD_600nm_ = 0.1 and incubated overnight at 37°C. Next, the preculture was diluted in LB medium supplemented with 50 μg/ml kanamycin to OD_600nm_ = 0.05. The culture was incubated at 37°C until the OD_600nm_ reached 0.6–0.8. Protein expression was induced with 0.2 mM IPTG and incubated for 5 h. Cells were harvested and kept at −80°C. Cells were resuspended in lysis buffer (40 mM Tris–HCl, 500 mM NaCl, 10 mM Imidazole, 1 mM PMSF, 0.2 mg/ml DNase1, 1× complete protease inhibitor cocktail (Roche), pH 7.4). Cell disruption was performed with an EmulsiFlex‐C3 (AVESTIN). The lysate was cleared by centrifugation in an SS‐34 rotor at 23,000 *g* at 4°C for 60 min. Next, the supernatant was collected and injected in two HisTrap columns (Cytiva) in tandem pre‐equilibrated in buffer A1 (40 mM Tris–HCl, 500 mM NaCl, 10 mM Imidazole, pH 7.4). After exhaustive washing with buffer A2 (40 mM Tris–HCl, 500 mM NaCl, 30 mM Imidazole, pH 7.4). Bound protein was eluted in a gradient 0–100% of buffer B (40 mM Tris–HCl, 500 mM NaCl, 500 mM Imidazole, pH 7.4). The fractions containing the protein of interest were pooled, and the buffer was exchanged with Desalting buffer (20 mM Tris–HCl, 150 mM NaCl, pH 7.4) in HiPrep™ 26/10 Desalting column (Cytiva). Protein concentration was determined, and an adequate amount of His_6_‐SUMO protease was added. The digestion was performed overnight at 4°C in the presence of 1 mM DTT and 5% of glycerol. Digestion efficiency was confirmed by SDS–PAGE. Next, imidazole 1 M pH 8.0 was added to the digestion mix to a final concentration of 20 mM. To deplete His_14_‐SUMO‐tag and the His_6_‐tagged SUMO protease, an appropriate volume of Protino^®^ Ni‐NTA‐Agarose (MACHEREY‐NAGEL) slurry was washed with Desalting buffer. Then, the digestion mix was added to the sedimented beads and incubated overnight at 4°C with end‐to‐end mixing. The unbound fraction was collected by gravity flow, and the bound protein was eluted with equivalent volume of buffer B. The quality of depletion was assessed by SDS–PAGE.

### Synthesis of protein‐DyLight fluorescent adducts

Purified proteins were reduced with TCEP (Sigma‐Aldrich). Excess TCEP was removed by buffer exchange to Maleimide buffer (100 mM potassium phosphate, 150 mM NaCl, 250 mM sucrose, 1 mM EDTA, pH 6.6) in HiPrep™ 26/10 Desalting column (Cytiva). To fluorescently label the proteins, reduced protein was mixed with a 3:1 molar excess of DyLight_488_/DyLight_680_/DyLight_800_‐maleimide (in dimethylformamide) and incubated overnight at 4°C. Unreacted maleimide groups were quenched with a 50‐fold molar excess of cysteine. Finally, the buffer was exchanged to 20 mM HEPES, 150 mM KCl, 5% glycerol, pH 7.4 in HiPrep™ 26/10 Desalting column (Cytiva). The protein concentration was determined and set to 0.5–1 mg/ml. Aliquots of the labeled protein were kept at −80°C until used.

### Mitochondrial isolation

Yeast mitochondria were isolated using differential centrifugation (Meisinger *et al*, 2006). YP Media (1% yeast extract, 2% peptone) containing 2% glucose (YPD for primary cultures) or 3% glycerol (YPG for secondary cultures) was used as carbon source to grow wild‐type YPH499 (MATa *ade2‐101*, *his3‐∆200*, *leu2‐∆1*, *ura3‐52*, *trp1‐∆63*, *lys2‐801*), BY4741 wild‐type (MATa *his3∆1*, *leu2∆0*, *met15∆0*, *ura3∆0*) and BY4741 *atp21*Δ yeast strains (Euroscarf) at 30°C with shaking till OD_600_ reached 1.5–2.5, after which they were harvested. The pellet was washed with water to remove remaining media and treated with DTT buffer (10 mM DTT, 100 mM Tris–HCl, pH 9.4) for 30 min at 30°C with shaking. Cells were washed with 1.2 M Sorbitol and treated with Zymolyase buffer (20 mM KPO_4_, pH 7.4, 1.2 M sorbitol, and 0.57 mg/l zymolyase) for 1 h at 30°C with shaking. Spheroplasts were harvested and resuspended in cold homogenization buffer (600 mM sorbitol, 10 mM Tris–HCl, pH 7.4, 1 g/l BSA, 1 mM PMSF, and 1 mM EDTA) before lysis using a homogenizer. Mitochondria were subsequently isolated using differential centrifugation and resuspended in SEM buffer. Protein concentration in the isolated mitochondria was determined using a Bradford assay and the final concentration adjusted to 10 mg/ml using SEM (250 mM sucrose, 20 mM MOPS/KOH pH 7.2, 1 mM EDTA) buffer before aliquoting and snap‐freezing for storage at −80°C.

Mutant strain *tim44‐804* (*MATa*, *ade2‐101*, *his3*‐Δ*200*, *leu2*‐Δ*1*, *ura3‐52*, *trp1*‐Δ*63*, *lys2‐801*, *tim44::ADE2* [*pBG‐TIM44‐0804*]) and the corresponding wild‐type cells were grown under permissive conditions, and mitochondria isolated as described previously (Hutu *et al*, 2008).

Mitochondria depleted in Tim50, the AG55Gal strain (*MAT*a, *ade2‐101*, *his3‐Δ200*, *leu2‐Δ1*, *ura3‐52*, *trp1‐Δ63*, *lys2‐801*, *tim50::HIS3‐PGAL1‐TIM50*) and the corresponding wild‐type cells were grown and processed as described previously (Schulz *et al*, 2011).

### Import into isolated mitochondria

For the synthesis of [^35^S] labeled Jac1 and Atp5 precursors, mRNA was generated using the mMessage mMachine SP6 Transcription Kit (Invitrogen), following the manufacturer's instructions. The mRNA obtained was used for *in vitro* translation in Flexi Rabbit Reticulocyte Lysate System (Promega), and the resulting lysate was directly used in the import reaction. The fluorescent precursors, as described above, were thawed just prior to import.

Import reactions were performed as described by Ryan *et al* (2001). Mitochondria were resuspended in import buffer (250 mM sucrose, 10 mM MOPS/KOH pH 7.2, 80 mM KCl, 2 mM KH_2_PO_4_, 5 mM MgCl_2_, 5 mM methionine, and 3% fatty acid‐free BSA) and supplemented with 2 mM ATP and 2 mM NADH (as well as 5 mM creatine phosphate and 1 μg/μl creatine kinase for experiments where the time for import exceeded 15 min). Import was performed by incubating samples at 25°C and stopped by the addition of 1% AVO (final concentration 1 μM valinomycin, 8 μM antimycin A, and 20 μM oligomycin). The import samples were treated with 20 μg/ml proteinase K (PK) for 10 min on ice. Samples were treated with 2 mM PMSF, incubated on ice for 10 min and centrifuged to sediment the mitochondria which were washed with SEM buffer and then analyzed by SDS–PAGE followed by Western blotting and digital autoradiography (Amersham Typhoon, Cytiva) or fluorescent scanning (Starion FLA‐9000, FujiFilm), based on the imported precursor. Signals were quantified using ImageQuant LT (GE Healthcare) with a rolling ball background quantification. Alternatively, for the 96‐well format, the mitochondria were centrifuged after proteinase K and PMSF treatment, washed with SEM, resuspended in SEM buffer for transfer to a 96‐well plate, and read on the Spark Multimode Microplate Reader (Tecan). Values of import were normalized by subtracting the signal from the AVO control sample and graphically represented using GraphPad Prism 8.

Mitochondria isolated from *tim44* temperature‐conditional yeast mutant were incubated at 37°C for 15 min in import buffer prior to the addition of ATP, NADH, and the precursor.

### Quantification of imported protein

Purified proteins were diluted in desalting buffer. A standard curve was plotted upon fluorescence measurement. This standard curve was used to calculate the corresponding total protein amount in the import samples.

### Membrane potential reduction—CCCP titration

Increasing amounts of the uncoupler CCCP were titrated into the import reaction to reduce the membrane potential (van der Laan *et al*, 2006). Import buffer used for the import reaction (as described above) was supplemented with 1% fatty acid‐free BSA and 20 μM oligomycin. Mitochondria were kept at 25°C for 5 min prior to precursor addition.

## Author contributions

**Naintara Jain:** Conceptualization; data curation; investigation; methodology; writing – original draft; writing – review and editing. **Ridhima Gomkale:** Conceptualization; data curation; investigation; writing – review and editing. **Olaf Bernhard:** Investigation. **Peter Rehling:** Conceptualization; supervision; funding acquisition; writing – original draft; writing – review and editing. **Luis Daniel Cruz‐Zaragoza:** Conceptualization; data curation; formal analysis; supervision; investigation; writing – original draft; writing – review and editing.

## Disclosure and competing interests statement

The authors declare that they have no conflict of interest.

## Supporting information



Expanded View Figures PDF

PDF+

## Data Availability

This study includes no data deposited in external depositories.
