# Supplementary material for: A quantitative fluorescence‐based approach to study mitochondrial protein import
Source: EMBO Rep. 2023 Mar 20;24(5):e55760. doi: 10.15252/embr.202255760 (PMC10157374; doi:10.15252/embr.202255760)
Supplement: Supplementary file 1 — Expanded View Figures PDF [file EMBR-24-e55760-s001.pdf]

Expanded View Figures

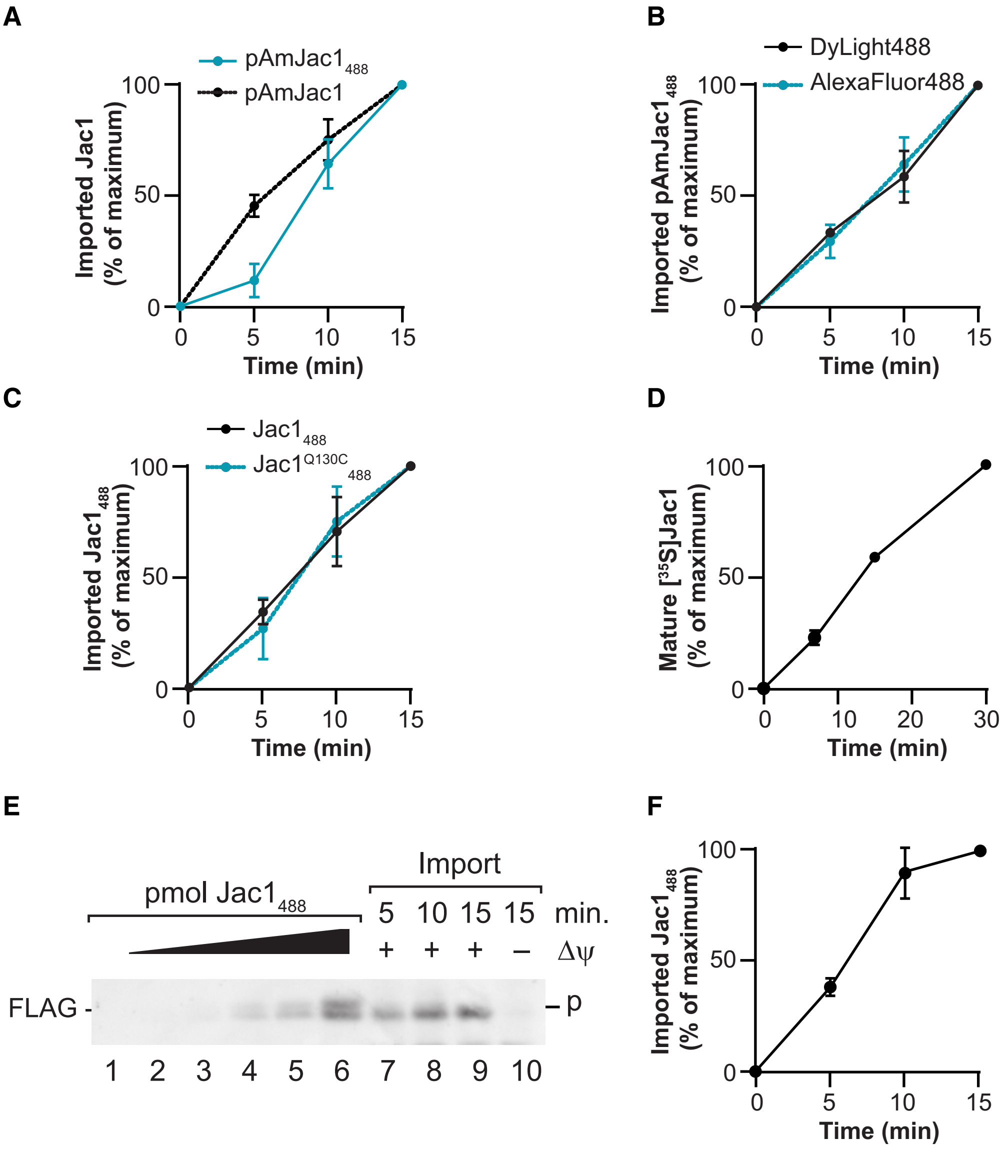

Figure EV1. Effect of precursor modification with a fluorophore on protein import.

A Quantification of import of pAmJac1 modified (pAmJac1$_{488}$) and non-modified (pAmJac1). The amount of imported protease-protected samples at 15 min was set to 100% in each case; error bars indicate SEM (*n* = 4).

B Quantification of import of pAmJac1 conjugated to DyLight488 or Alexa Fluor 488. The amount of imported protease-protected samples at 15 min was set to 100% in each case; error bars indicate SEM (*n* = 3).

C Quantification of import of Jac1 modified with DyLight488 at the C-terminus (Jac1$_{488}$) and internally (Jac1$^{Q130C}{}_{488}$). The amount of imported protease-protected samples at 15 min was set to 100% in each case; error bars indicate SEM (*n* = 3).

D Quantification of import of radioactive [^{35}S]Jac1. The amount of imported protease-protected samples at 30 min was set to 100%; error bars indicate SEM (*n* = 3).

E Immunoblot-based analysis of Jac1$_{488}$ import into mitochondria from Fig 1C. The PDVF membranes were incubated with anti-FLAG antibody for Jac1 detection.

F Quantification by immunoblot-based detection of Jac1$_{488}$ import shown in (E). The amount of imported protease-protected samples at 15 min was set to 100%; error bars indicate SEM (*n* = 3).

Data information: The values represented in the graphs correspond to the arithmetic mean ± standard error of the mean (SEM). The number of biological independent replicates (*n*) for each experiment is indicated for each assay.

© 2023 The Authors

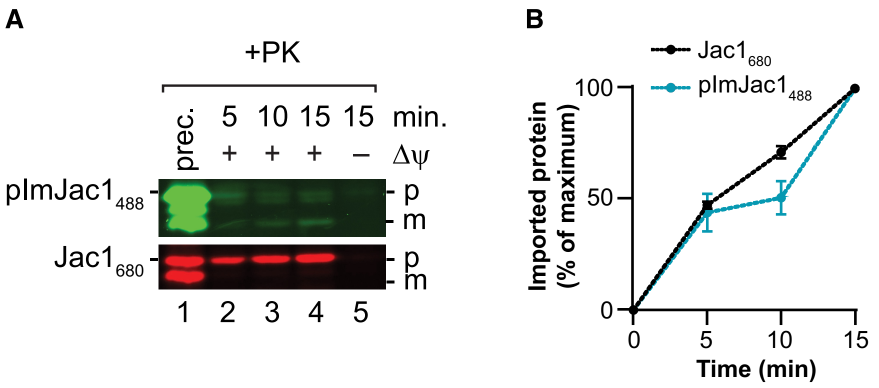

Figure EV2. Parallelization of import.

A Co-import of $Jac1_{680}$ and $pImJac1_{488}$ into wild-type mitochondria for indicated times. Samples were treated with Proteinase K. Prec., purified precursor protein; p, precursor; m, mature.

B Quantification of co-import shown in (A). The amount of imported protease-protected samples at 15 min was set to 100% in each case; error bars indicate SEM (n = 3).

Data information: The values represented in the graphs correspond to the arithmetic mean ± standard error of the mean (SEM). The number of biological independent replicates (n) for each experiment is indicated for each assay.

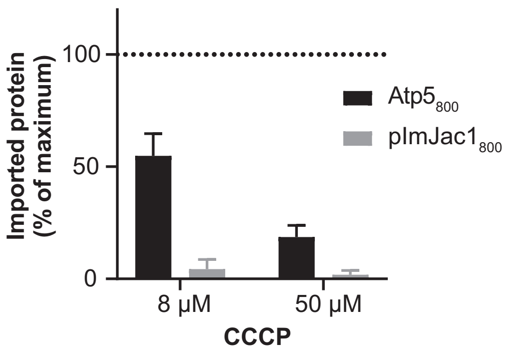

Figure EV3. Membrane potential influence over the import of different precursors.

Quantification of import of $Atp5_{800}$ and $pImJac1_{800}$ over 15 min into mitochondria treated with 8 and 50 µM CCCP. The amount of imported protease-protected protein in the absence of CCCP was set to 100%; error bars indicate SEM (n = 3).

Data information: The values represented in the graphs correspond to the arithmetic mean ± standard error of the mean (SEM). The number of biological independent replicates (n) for each experiment is indicated for each assay.

© 2023 The Authors
